# First report of hare treponematosis seroprevalence of European brown hares (*Lepus europaeus*) in the Czech Republic: seroprevalence negatively correlates with altitude of sampling areas

**DOI:** 10.1186/s12917-019-2086-3

**Published:** 2019-10-18

**Authors:** Markéta Nováková, David Najt, Lenka Mikalová, Marcela Kostková, Eliška Vrbová, Michal Strouhal, Annika Posautz, Sascha Knauf, David Šmajs

**Affiliations:** 1 Department of Biology, Faculty of Medicine, Masaryk University, Kamenice 5, Building A6, 625 00 Brno, Czech Republic; 2 Department of Infectious Diseases and Microbiology, Faculty of Veterinary Medicine, University of Veterinary and Pharmaceutical Sciences Brno, Brno, Czech Republic; 3 State Veterinary Institute Jihlava, Jihlava, Czech Republic; 4 Research Institute of Wildlife Ecology, University of Veterinary Medicine Vienna, Vienna, Austria; 5 Work Group Neglected Tropical Diseases, Infection Biology Unit, German Primate Center, Leibniz Institute for Primate Research, Goettingen, Germany; 6 Division of Microbiology and Animal Hygiene, Georg-August-University of Goettingen, Goettingen, Germany

**Keywords:** Hare disease, Venereal disease, Wildlife disease, Game animals, Lesion, *Lepus europaeus*, *Lepus timidus*, Lagomorphs, *Treponema paraluisleporidarum*, Treponematosis

## Abstract

**Background:**

The aim of this study was to quantify the seroprevalence of hare treponematosis in European brown hare (*Lepus europaeus*) populations in the Czech Republic and to test for an association between treponematosis prevalence and the altitude of the areas in which hares were sampled. We tested 289 serum samples of brown hares collected between 2015 and 2017. The sampling areas included 12 districts (73 villages) distributed throughout the Czech Republic. Serum samples were tested for the presence of antibodies against the causative agent of hare treponematosis (*Treponema paraluisleporidarum* ecovar Lepus, TPeL) using two serological tests for human syphilis that cross-react with TPeL: the *Treponema pallidum* hemagglutination assay (TPHA) and the fluorescent treponemal antibody absorption (FTA-ABS) test. To account for the imperfect diagnostic sensitivity and specificity of each test, apparent prevalence estimates of TPeL were converted to true prevalence estimates using the Rogan Gladen estimator.

The correlation between TPeL true seroprevalence and altitude of sampling areas was analyzed using Pearson’s correlation coefficient at three levels of spatial resolution: (1) four groups, each composed of two merged districts, with ≥20 samples collected, differing in their altitude median (206, 348, 495, and 522 m above sea level); (2) separately tested eight districts, where ≥20 samples were collected per district; and (3) 27 groups composed of villages of the same altitude level distributed across the whole dataset.

**Results:**

One hundred and seven of the 289 samples were seropositive to both tests, the FTA-ABS test was positive for an additional 47 samples. Seropositive samples were found in all 12 districts. True seroprevalence of TPeL in the sampled hares was 52% (95% confidence interval 46 to 58%).

A statistically significant negative correlation between TPeL seroprevalence and altitude was identified at the district level (Pearson’s *r* = − 0.722, *p* = 0.043).

**Conclusions:**

Between 2015 and 2017 hare treponematosis was present at a relatively high prevalence in brown hares in all 12 districts in the Czech Republic where sampling was carried out. The seroprevalence of TPeL in brown hares was negatively correlated with the altitude of the areas in which hares were sampled.

## Background

The bacterial genus of *Treponema* is comprised of both nonpathogenic and pathogenic species, some of which cause important human and animal diseases [1, 2]. The causative agents of human syphilis (*T. pallidum* subsp. *pallidum*, TPA), yaws (subsp. *pertenue*) and bejel (subsp. *endemicum*) show minimal genetic differences with their closest relatives *Treponema paraluisleporidarum* ecovar Cuniculus (TPeC) and ecovar Lepus (TPeL) in rabbits and hares, with sequence identities of greater than 98% [2, 3].

TPeC and TPeL cause syphilis-like infections in lagomorphs. The first description of TPeC was in 1920 in rabbits (*Oryctolagus cuniculus*) [4]. The pathogen was initially named *Spirochaeta paralues-cuniculi*, however, it was later reclassified as TPeC [5]. TPeC causes a sexually transmitted infection that is characterized by crusting ulcers in the anogenital region, nose, eyelids, lips, and paws [6]. The infection can be transmitted from mother to neonates intrapartum while transplacental transmission, as seen with human syphilis, has not been demonstrated [7].

In contrast to TPeC, TPeL infection was first described in European brown hares (*Lepus europaeus*) and mountain hares (*Lepus timidus*) in 1957 [8]. Although most animals infected with TPeL show no signs of disease [9] some develop orofacial and anogenital proliferative crusty skin lesions at mucocutaneous junctions. The presence of TPeL serum antibodies in samples taken from trapped hares has been confirmed in seven European countries with apparent seroprevalence estimates ranging from 1 to 64% [7, 8, 10–13]. The aim of this study was to quantify the seroprevalence of TPeL in European brown hare populations in the Czech Republic and to test for an association between treponematosis prevalence and the altitude of the areas in which hares were sampled.

## Results

In total, 107 out of 289 sera samples tested positive for TPeL using the *T. pallidum* hemagglutination assay (TPHA) and 154 out of 289 sera samples tested positive using the fluorescent treponemal antibody absorption (FTA-ABS) test. Forty-two (14%) samples, non-evaluable using the TPHA (i.e. reactive with fowl erythrocytes without treponemal antigens), were distributed equally through all result categories (from 4+ to 1+) of FTA-ABS (Additional file 1: Table S1). From the non-evaluable samples, 10 were excluded due to hemolysis and the remaining 32 were retested after pre-absorption, resulting in six positive, 13 negative, and 13 non-evaluable samples. The 10 hemolytic samples and the 13 non-evaluable samples were excluded from further analyses. None of the samples that were TPHA-positive tested FTA-ABS-negative (Additional file 1: Table S1). For the FTA-ABS test, 29% of samples reacted as 4+, 12% as 3+, 7% as 2+, and 5% as 1+. Interpreting the two test results in parallel and accounting for the imperfect diagnostic test sensitivity and specificity of each test using the Rogan Gladen estimator, the true prevalence of TPeL in European brown hare populations in the Czech Republic was estimated to be 52% (95% CI 46 to 58%).

**Fig. 1:**
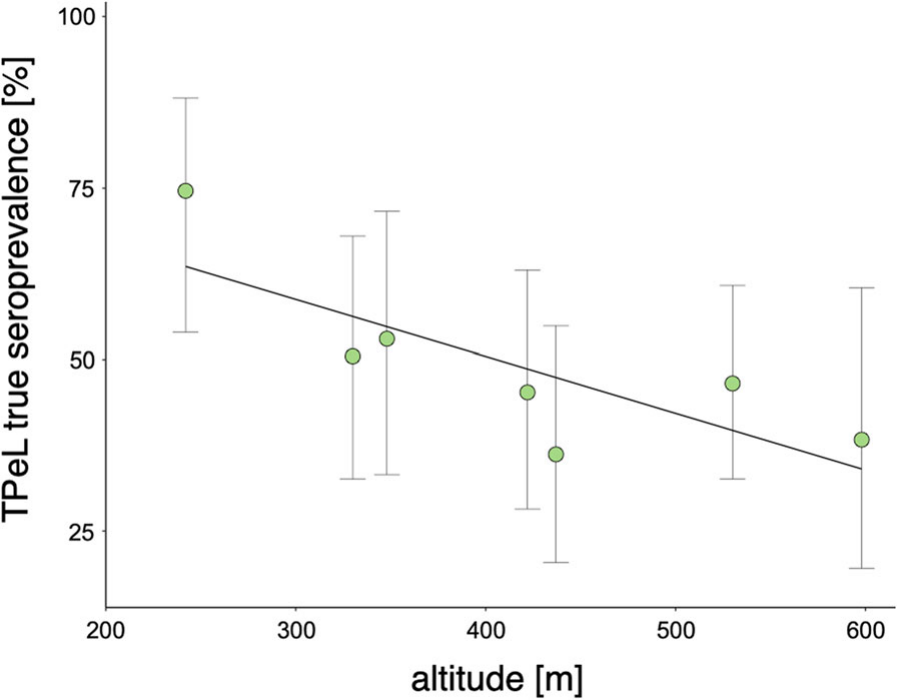
Error bar plot showing the estimated true prevalence of hare treponematosis as a function of median elevation in eight Czech districts

**Fig. 2:**
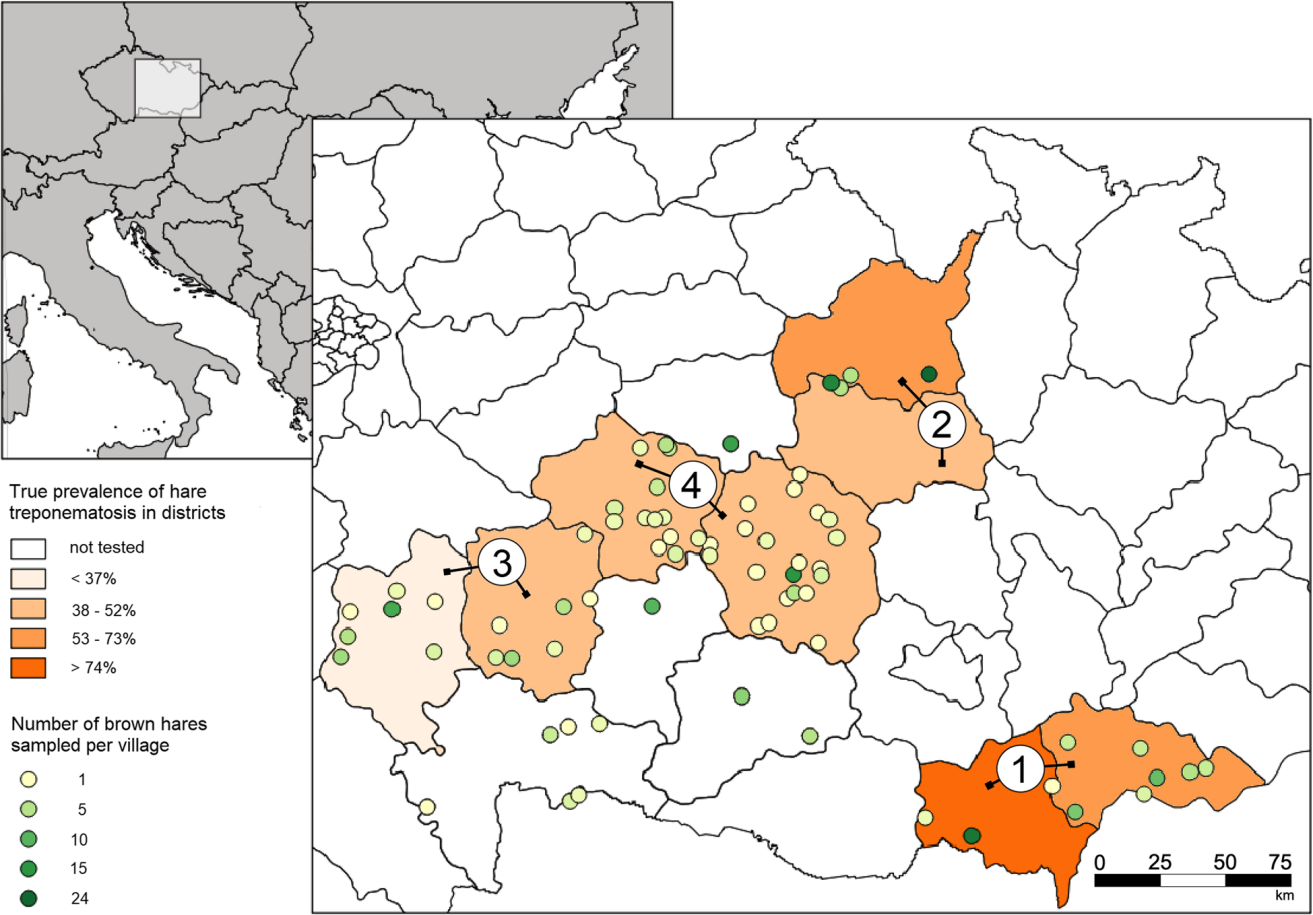
Choropleth map showing the estimated true prevalence of hare treponematosis in brown hares in Czech Republic districts, 2015 to 2017. Superimposed on each plot are points showing the number of hares sampled in villages within each district. Districts with 20 or more samples were merged into four regions differing in altitude median (group 1: Břeclav + Hodonín, 206 m above sea level; group 2: Svitavy + Ústí nad Orlicí, 348 m above sea level; group 3: Pelhřimov + Tábor, 495 m above sea level; group 4: Havlíčkův Brod + Žďár nad Sázavou, 522 m above sea level). The depicted map was prepared in open source geographic information system QGIS by the authors of this study

We identified a statistically significant negative correlation between TPeL seroprevalence and the altitude of the district in which hares were sampled (Pearson’s *r* = − 0.722, *p* = 0.043) (Fig. 1). The analysis of the largest areas, i.e., four groups differing in altitude (Fig. 2), showed a similar but statistically insignificant trend (Pearson’s *r* = − 0.907, *p* = 0.093).

## Discussion

Our study shows that between 2015 and 2017 relatively high proportions of brown hares in the Czech Republic were seropositive to the causative agent of hare treponematosis, *Treponema paraluisleporidarum* ecovar Lepus. TPeL seroprevalence was assessed using two treponemal tests: the TPHA and the FTA-ABS test.

The TPHA is widely used in human healthcare settings with a diagnostic sensitivity for detection of syphilis of > 95% and a diagnostic specificity of > 99% [14]. Since some specimens may be non-evaluable, the FTA-ABS test (with a diagnostic sensitivity of 90.8% and diagnostic specificity of 98%) has been proposed as a confirmatory test for human syphilis [15, 16]. In this study, the TPHA was performed according to original protocol (IMMUTREP®, Omega Diagnostics LTD., United Kingdom), and the FTA-ABS test was optimized for detection of TPeL in brown hares using a secondary anti-hare antibody. There was a substantially smaller number of test-positive samples using the TPHA (107 out of 289) compared with the FTA-ABS test (154 out of 289). None of TPHA-positive samples returned a negative result when tested using the FTA-ABS test. Interpreting the two sets of test results in parallel increased the diagnostic test sensitivity to 99.5% and decreased the diagnostic specificity to 97%.

A limitation of our study was the availability of the amount of anti-hare antibody, which was sufficient for screening of 289 samples instead of 435, which would have allowed us to be 95% confident that our estimate of the seroprevalence of TPeL was within 0.05 of the true population value. Based on 289 samples we can be 95% confident that our estimate of the true prevalence of TPeL was within 0.06 of the true population value.

An additional limitation of our study was the exclusion of tularemia-positive samples, which could have biased our results, since coinfections can theoretically occur, with TPeL-infected hares being more susceptible to tularemia.

Our correlation analyses identified a negative association between the true seroprevalence of TPeL and the altitude of the areas in which hares were sampled (Fig. 1, Additional file 2: Table S2). Aggregating the data to the district level yielded a greater number of samples per comparison group, providing greater statistical power to detect an association between TPeL seroprevalence and altitude. One possible explanation for our findings is that larger areas (i.e. districts) are occupied by spatially distinct brown hare populations. When samples were aggregated to smaller spatial areas a trend in TPeL seroprevalence and altitude was still present, although not statistically significantly so. This may have been because hares sampled in some location may have been placed at locally extremely high or low altitudes that do not represent the overall character of an area (e.g. a solitary hill in the middle of lowlands).

Altitude affects numerous abiotic and biotic factors (e.g. annual average temperature and the presence of specific vegetation communities). In the Czech Republic, areas below 300 m above sea level (group 1) are characterized by a higher proportion of suitable habitat areas (i.e. large open crop fields and vineyards) and higher brown hare densities. This contrasts to the hilly regions represented by groups 2, 3 and 4 which are composed of heterogeneous smaller fields separated by multiple forests that may restrict interaction between hare groups. Brown hare locomotor behaviour was found to be more localized in smaller fields [17], which may lead to less contact (e.g. mating opportunities) of polygamic brown hares in such areas and less opportunities for TPeL to spread among individuals [18]. Interestingly, a negative correlation with altitude was also found for tularemia in brown hares in the Czech Republic, where animals in the lowlands (where brown hare populations reach higher densities) were more frequently tularemia-infected [19]. In contrast to highly host specific TPeL, tularemia is known to infect and persist in a broad host spectrum and even in the environment. Since treponematoses are typically transmitted exclusively by direct contact between individuals, we speculate that TPeL infections may depend on hare density to a greater extent than tularemia.

In comparison with countries where the presence of hare syphilis has been previously quantified, the TPeL seroprevalence reported in this study was similar to the apparent TPeL seroprevalence found in Germany, where it reached 43 and 44% in two distant populations of 28 and 41 European brown hares tested using the FTA-ABS test [7]. The study of Posautz and colleagues [7] was restricted to a limited geographical area of Germany including one island and one mainland region. Moreover, Posautz and colleagues found that seropositivity was greater in adult females compared with subadults and males. In contrast, no significant difference between sexes was found in 154 brown hares sampled from seven areas in Central Italy, where 33% of samples were TPHA positive. Lack of information about the sex and age of sampled hares was a limitation of our study. In Hungary, 28% hares were found to be rapid plasma reagin positive [10]. When 71 out of 202 samples were randomly selected by Horvath et al. [10] and tested using the Kolmer’s complement fixation reaction with Reiter protein, positivity reached 66%, which is similar to the seroprevalence estimates reported in this study. In Italy, a significantly higher seroprevalence was found in areas with high population densities of hares, which corroborates with our findings [13].

Hare treponematosis has been found to infect both brown hares (0.9%) and mountain hares (3%) in Sweden, where 1118 and 760 samples were tested, respectively [11]. The effect of climate on brown hare densities is complex, but temperature has been found to be positively correlated with brown hare abundance and also with infection rates of several infectious diseases [20].

Information on the occurrence of lesions in the hares that were sampled for this study was not available; however, the presence of lesions in TPeL-positive hares are rarely observed [10, 12]. Although the mortality rate of hare treponematosis is unknown, lesions may reduce the fitness of the animal [11] or could serve as an entry point for secondary bacterial infection. It appears that hare syphilis remains clinically inapparent or not evident in most cases since animals with lesions are rarely reported. The precise proportion of inapparent and clinically apparent infections is unknown.

While TPA causes both orchitis and intradermal lesions in rabbits, hares are only moderately susceptible to TPA since intratesticular inoculation leads to orchitis, but after intradermal inoculation lesions fail to develop within 4 weeks [10]. While TPeL induces orchitis in both rabbits and hares, TPeC results in orchitis only in rabbits [5]. Since TPeC seems to have fewer hosts than TPeL, TPeL may be an evolutionary ancestor of TPeC [21]. Further studies will be needed to identify the phylogenetic relatedness of TPeL to other pathogenic treponemes.

A continuous decline in hare population has been observed since the 1960s together with a continuous reduction in rural species richness and abundance in the agricultural landscapes of Europe [22]. Agricultural intensification and climate change are thought to be two of the main reasons for this decline [11, 20]. Although hare syphilis may contribute to decreases in the number of hares, the exact contribution of hare treponematosis to the ongoing decline of the European hare population remains unknown.

## Conclusions

We identified a relatively high seroprevalence of treponematosis among brown hares sampled in the Czech Republic between 2015 and 2017. The seroprevalence of TPeL in brown hares was negatively correlated with the altitude of the areas in which hares were sampled. Since altitude affects numerous abiotic and biotic factors including temperature and the presence of vegetation communities, further studies are needed to elucidate which conditions favor the transmission of TPeL among brown hare populations.

## Methods

A total of 1813 serum samples from European brown hares were obtained from 12 districts in the Czech Republic. Samples were collected during the hare hunting season which occurs in November, December, and January (between November 2015 and January 2017). Hares were shot by registered hunters and blood was collected post mortem from the heart without anticoagulants. All samples were submitted for mandatory tularemia surveillance at the State Veterinary Institute Jihlava prior to this study. Tularemia-positive samples (*n* = 121; 7%) were excluded from further analyses. From the remaining 1692 tularemia-negative samples a random selection of samples was performed by assigning a random number to each sample identifier using the random number generator function in Microsoft Excel. The list of sample identifiers and their assigned random numbers were sorted in order of the random number and the first 289 samples selected for analysis. A total of 435 samples were required, assuming the true prevalence of TPeL was 50% and that we wanted to take a sufficient number of samples to be 95% confident that the estimated prevalence of TPeL was within 5% of the true population value using a test with diagnostic sensitivity of 99% and diagnostic specificity of 97% [23]. Since the limited amount of secondary antibody available allowed testing of 289 samples, we could be 95% confident that our estimate was within 6% of the true population value.

Samples were tested for the presence of anti-TPeL antibodies by using commercially available treponemal tests established for human syphilis and cross-reacting with TPeL. First, we utilized the TPHA (IMMUTREP®, Omega Diagnostics LTD., United Kingdom) where fowl erythrocytes, coated with antigenic components of TPA strain Nichols (Test Cells), agglutinate in the presence of anti-treponemal antibodies. Serum sample aliquots (25 μL) were serially diluted in the diluent to 1:19. Next, 25 μL of this solution was mixed with 75 μL of Test Cells, or non-TPA antigen coated erythrocytes (Control Cells), which resulted in a final sample dilution of 1:80. Agglutination patterns were examined after 60 min of incubation at room temperature. Nonspecific reactions were identified during testing with Control Cells and were subsequently retested following pre-absorption of sera diluted 1:4 in reaction with Control Cells, incubation for 60 min at room temperature and centrifuging for 5 min at 1000 rpm. The resulting supernatant was diluted 1:5 in the diluent and retested for specific anti-treponemal antibodies.

As a confirmatory test, we used the FTA-ABS (MASTAFLUOR™, Mast Diagnostica GmbH, Germany). This test uses whole TPA strain Nichols bacteria, which have been immobilized on a glass slide. Serum antibodies that bind to TPA outer membrane components were detected using fluorescein isothiocyanate (FITC)-labeled secondary anti-hare immunoglobulin G, which was prepared in rabbits, at a dilution of 1:400 in PBS. Serum samples (10 μL) were diluted 1:5 in Sorbent (culture extract of *Treponema reiteri*), incubated at 37 °C for 30 min, then diluted 1:8 and 1:64 in PBS. Next, 25 μL of a treated specimen was placed on a glass slide, incubated at 37 °C for 30 min, washed in PBS, covered with 25 μL of FITC-labeled anti-hare conjugate, incubated at 37 °C for 30 min and finally washed in PBS.

The positive and negative control for the TPHA test were included in the test kit; sample 20,775/4 (Additional file 1: Table S1), which reacted strongly positive in both dilutions, was chosen as the positive control for the FTA-ABS tests. The Absorption Control (human serum with nonspecific antibodies to treponemes) diluted 1:5 in Sorbent was provided in the kit. Results of the FTA-ABS tests were evaluated as strongly or weakly positive in both dilutions compared to the positive control (denominated as 4+, 3+, 2+, 1+). Results of the two tests were interpreted in parallel and the true prevalence of TPeL was estimated according to the Rogan and Gladen method [24, 25].

The correlation between the estimated true seroprevalence of TPeL and the altitude of the areas in which hares were sampled was assessed using Pearson’s correlation coefficient [26]. Analyses were carried out at three levels of spatial resolution: (1) four groups, each composed of two merged districts, with more than 20 samples collected, differing in their altitude median (group 1: Břeclav and Hodonín, 206 m above sea level; group 2: Svitavy and Ústí nad Orlicí, 348 m above sea level; group 3: Pelhřimov and Tábor, 495 m above sea level; group 4: Havlíčkův Brod and Žďár nad Sázavou, 522 m above sea level); (2) separately tested 8 districts, where more than 20 samples were collected per district; and (3) 27 groups composed of villages of the same altitude level distributed across the whole dataset (Additional file 1: Table S1). Statistical analyses were carried out using R version 3.5.2 [27]. The Geographic Information System Quantum GIS was used for mapping [28].

## Supplementary information

**Additional file 1: Table S1.** A list of European brown hares (*Lepus europaeus*) sampled in the Czech Republic, 2015 to 2017, that were tularemia-negative. Hares were tested for the presence of hare treponematosis by the TPHA and FTA-ABS tests. Abbreviations: a.s.l.: above sea level; non: non-evaluable; P: positive; N: negative; 4+: strongly reacting in both dilutions; 3+: strongly reacting in 1:8 dilution, weakly reacting in 1:64 dilution; 2+: weakly reacting in both dilutions; 1+: weakly reacting solely in 1:8 dilution.

**Additional file 2: Table S2.** The correlation between true seroprevalence of TPeL and altitude median estimated at three levels: (1) grouped districts; (2) districts; (3) villages grouped according to the altitude. Eight out of 12 districts (for grouped districts and districts) and 27 out of 59 altitude levels (i.e. 39 out of 73 villages) were selected for the correlation analyses, the criterion was ≥20 and ≥ 4 of samples available, respectively. A negative correlation (*r* < − 0.5) between estimated true seroprevalence of TPeL and altitude median was observed at the district level. Abbreviations: No: number; CI: confidence interval; *r*: Pearson correlation coefficient; p: *p* value; BV: Břeclav; HB: Havlíčkův Brod; HO: Hodonín; PE: Pelhřimov; SY: Svitavy; TA: Tábor; UO: Ústí nad Orlicí; ZR: Žďár nad Sázavou.

## Data Availability

All data generated or analysed during this study are included in this published article and its supplementary information files.

## Abbreviations

FTA-ABS: Fluorescent treponemal antibody absorption
TPA: *Treponema pallidum* subsp. *pallidum*
TPeC: *Treponema paraluisleporidarum* ecovar Cuniculus
TPeL: *Treponema paraluisleporidarum* ecovar Lepus
TPHA: *Treponema pallidum* hemagglutination assay
